# An Essay on Pneumonia, Read before the Atlanta Medical Society

**Published:** 1856-02

**Authors:** Thos. M. Darnall


ATLANTA
Medical and Surgical Journal.

Vol. I.] FEBRUARY, 1856. [No. 6.

### ORIGINAL COMMUNICATIONS.

### ARTICLE I.

*An Essay on Pneumonia, read before the Atlanta Medical Society.* By Thos. M. Darnall, M. D., Dec. 6, 1855. (Published by the Society.)

Mr. President:

The frequent occurrence of this disease in our climate, must be my apology for inviting your attention to its consideration this evening. Very few of our fraternity, who have been engaged in practice any length of time, have been fortunate enough to escape a contest with this fierce enemy of human life.

Sometimes it makes its approaches stealthily, as when it originates from an ordinary "bad cold," or common catarrh; but it usually comes on suddenly—the patient complains of pain, more or less, acute in some part of the thorax, with a sense of fullness or stifling; respiration is hard—the pulse frequent, and hard generally; but sometimes it is frequent and soft, especially when a large portion of one, or both lungs are involved in the disease.

The countenance is expressive of anxiety, in proportion to the gravity of the attack. The features are frequently full, at the beginning of the disease. The cheeks are more or less flushed with a circumscribed spot on each—sometimes on one cheek only—resembling precisely, the flush we so frequently see on the cheeks of the victims of pulmonary consumption.

The tongue is more or less furred and moist, at the beginning, but early in the course of the disease, it becomes dry and red, or brown in the middle, and red at the tip and edges.

When the attack is severe, this organ appears to be slightly swollen, and livid—the lips also assume the same unnatural hue. The cough which usually attends an attack of Pneumonia, aggravates the pain, and dyspnœa continues throughout the whole course of the disease. Occasionally the cough is absent, for the first two or three days, in very severe cases. Expectoration is always difficult at first, and generally tinged with blood, becoming more free in a few days, with less difficulty of breathing.

When the inflammation, instead of passing off by resolution, runs into suppuration, rigors are experienced—the respiration becomes more oppressed, and a sense of weight is felt in the side where the matter is lodged.

Pneumonia does, sometimes, terminate in gangrene; but this must be very rare. I have seen it only once. Hepatization is not unfrequent; indeed, in every post mortem examination that I have witnessed of those who have died of Pneumonia, the lung, or lungs were found in this condition, to a greater or less extent.

If a sufficient number of the symptoms already enumerated, should fail to be present in any case, to enable us to make a satisfactory diagnosis, we may resort to percussion and auscultation. In the first stage, or stage of engorgement, a dull sound will generally be given by the affected part, from percussion; auscultation will, with equal certainty, show a dry crepitating rale. The respiratory murmur is intermingled with crepitation generally, but it is sometimes absent.

Hepatization is known by the dullness of the sound given out from percussion, in every position of the body of the patient—the impeded motion of the affected side, and by the depression of the triangular space above the sternum of the corresponding side. Auscultation reveals the bronchial sound, instead of the crepitation which existed in the preceding stage. The respiratory murmur is louder in the portion of the lung not affected, and the action of the heart is more distinct. The third, or suppurative stage, is marked by pretty much the same signs, to which a coarse mucous rale may be added.

Those who may have profited by the lessons of experience and observation, will always give a guarded prognosis, because it is a disease that cannot be trifled with safely, and many cases, in spite of our best directed efforts, will terminate fatally.

I am aware that a difference of opinion exists among the great luminaries of our profession, as regards the most successful plan of treatment to be adopted, to arrest the progress of the disease, or conduct the patient safely through it.

Some of our deservedly distinguished European brethren maintain that the lancet is not a very potent therapeutical agent in restraining the progress, or abbreviating the duration of the disease. Whatever may be their opinions, from the facts observed by them, I feel constrained, from my knowledge of its powers, skilfully and fearlessly employed, to place it in the front rank of our therapeutic agents, whenever we have acute inflammation to combat in any of the organs or tissues of the animal organism. All the phenomena characteristic of Pneumonia—fever, pain, excited circulation, with a hard pulse, &c., &c.—indicate its use, with a boldness proportioned to the violence of the attack. This should be done early, before any irreparable mischief has been sustained by the suffering organ. If we fail to diminish the volume of the circulating fluids sufficiently at the onset, or during the first stage, it will be too late when hepatization, suppuration, or gangrene has supervened. We would then only hasten the final catastrophe by its use, and bring distrust on ourselves, and odium on this invaluable agent.

But it may be asked, "would you not prostrate your patient by bleeding him freely, and induce a degree of debility from which he could not recover?" I reply, that free depletion, in the early stage of acute inflammation, is almost always, if not constantly, followed by healthy reaction. It is at least reasonable to expect such a result. All the symptoms attending acute inflammation, indicate clearly enough to the Medical Philosopher, that the circulatory apparatus is over tasked. The action of the heart is increased in force and frequency—the arterial system contracts forcibly on the vital current; this is indicated by the hardness of the pulse, and the turgidity of the veins. What is the inevitable result of this condition of the sanguiferous system if it is suffered to remain any length of time? Spontaneous hemorrhage, effusion or disorganization. Every practitioner who is at all conversant with the treatment of inflammatory diseases, will readily admit that the lancet, properly used, is more to be depended on than any other remedial agent with which we are acquainted, to prevent the terminations alluded to above.

I am free to admit, that all the tissues, when inflamed, are not equally affected by the loss of blood. This may, as is generally supposed, be owing to their relative remoteness from the heart. The mucous and fibrous tissues are not so readily relieved of inflammatory action, by the loss of blood, as the serous and muscular tissues. The knowledge of this fact should not induce us to neglect the use of this powerful agent, in the treatment of acute inflammation in any of the tissues, however remote, relatively, from the great centre of the circulation.

If the great enemy of life assails us at a remote point, and we cannot reach him with "drap and pill," why not boldly assail him with the tempered steel, and drive him from his fastnesses? Shall we let the fear of dangerous debility drive us from the use of this powerful agent? Can *it* be more dangerous than *debility*, resulting from hepatization or suppuration? I think not. We cannot determine *a priori*, the quantity of blood proper to be drawn at once from a patient. A very safe rule, is to allow the blood to flow from a *free* orifice as long as the patient can bear to lose it, in a sitting posture. It is not always necessary to allow the blood to flow until syncope is induced. If the pain and sense of fullness is entirely relieved, and the respiration is entirely free, we may arrest the flow, being assured that the patient has experienced the benefit of the remedy. Cases are occasionally met with that require a repetition of the remedy several times in the course of the disease; indeed, it is sometimes necessary to repeat the bleeding in an hour or two after the first operation. Nothing short of relieving the patient of pain and dyspnœa, should satisfy us, because, while these symptoms remain we may be sure that inflammation is doing its work, and that symptoms will soon arise that will spread a thick gloom over the hope of his physician and alarm his friends.

After a full and sufficient bleeding from the arm, Calomel, grs. v to x and Pulv. Doveri, grs. x to xv, repeated every three or four hours, I have found very beneficial. If the stomach is irritable, I usually combine Morph. Sulph., gr. ¼ to ½ with the Calomel. Either of these combinations generally procures rest for the patient, and a respite from all the most harassing symptoms. If practicable, this combination should be continued until free expectoration is established, or until the mercurial fœtor is clearly recognized on the breath of the patient.

In most of the cases I have treated for the last several years, I have alternated the antimonial solution, or antimonial wine, with the powders above spoken of, with satisfactory results. More recently, I have used Norwood's Tr. Verat Viride in the same way. It has so far, in my hands, proved an excellent expectorant, and controls the action of the heart with more certainty and to a greater extent, than any other medicine with which I am acquainted, that can be used with equal safety.—As soon as the mercurial fœtor is clearly establishd, I advise a discontinuance of the Calomel and go on with the antimonial preparation or the Verat. Viride, as long as the symptoms indicate a necessity for their use. Cupping, leeching and blistering over the chest, I am very much inclined to believe, are now, and always have been, greatly overrated in treating *Pneumonia.* I have seen blisters used advantageously, when applied to the extremities. When used on the arms and legs, their effect as revelants, may, I think, be much more reasonably expected. Applied immediately over these at of inflammation, they frequently fail to operate as revelants, and probably do harm by inviting a larger share of the circulating fluids to the vicinity of the part affected with disease.

Much has been said and written of the value of certain expectorant medicines, in the treatment of Pneumonia. They are most valuable in cases that have been inefficiently treated at the onset, or not treated at all until the disease has had time to entrench itself deeply in the pulmonary tissue. Of this class of remedial agents, the terebinthinates stand highest in my estimation, from the fact, that when used freely, they induce more or less irritation in the urinary apparatus, and in that way, may operate to some extent as revelants. The intimate sympathy known to exist between the viscera of the pelvis and the respiratory apparatus, has long since convinced Physicians and Surgeons of the impropriety of operating for the cure of Fistula in Ano, in patients laboring under or threatened with chronic pulmonary disease. But under more favorable circumstances, the conservative powers of the animal system—the "*vis medicatrix naturæ*"—aided by an abstemious diet, is all that is required to insure a restoration to health.

If the disease, from any cause, has been protracted, and the strength of the patient greatly reduced, then wine, brandy, and the vegetable and mineral tonics may be used with advantage.

